# Supplementary material for: Trends in Outcomes of Major Intracerebral Haemorrhage in a National Cohort of Very Preterm Born Infants in Switzerland
Source: Children (Basel). 2023 Aug 19;10(8):1412. doi: 10.3390/children10081412 (PMC10453192; doi:10.3390/children10081412)
Supplement: Supplementary file 1 [file children-10-01412-s001.zip › Thwaites et al. Table S1.pdf]

Table S1: Trend of outcomes of very preterm infants with grade 3 intraventricular haemorrhage (G3-IVH) and periventricular haemorrhagic infarction (PVHI) in Switzerland between 2002 and 2014.

**N (%), mean (SD)**	**2002**	**2003**	**2004**	**2005**	**2006**	**2007**	**2008**	**2009**	**2010**	**2011**	**2012**	**2013**	**2014**	**Total**	**Z [a] / β [b]**	**p-*value***
**Total live births**	322	301	362	349	371	361	377	397	428	441	420	389	438	4956	N.A.	N.A.
**G3-IVH**	8 (2%)	13 (4%)	10 (3%)	12 (3%)	21 (5%)	18 (5%)	9 (2%)	15 (3%)	9 (2%)	14 (3%)	16 (3%)	11 (2%)	16 (3%)	172/4956 (3%)	0.17 [a]	.103 [a]
- Gestational age	28.1 (1.5)	26.9 (1.8)	26.8 (1.1)	27.1 (1.4)	27.1 (1.3)	27.0 (1.6)	27.3 (1.3)	27.2 (1.7)	26.9 (1.7)	26.7 (1.4)	26.0 (0.9)	26.9 (1.9)	26.5 (1.4)	-	- 0.18 [b]	.020 [b]
- Death	3/8 (37%)	6/13 (46%)	4/10 (40%)	2/12 (17%)	7/21 (33%)	7/18 (39%)	3/9 (33%)	2/15 (13%)	1/9 (11%)	5/14 (36%)	5/16 (31%)	5/11 (45%)	6/16 (38%)	56/172 (33%)	0.19 [a]	.845 [a]
Withdrawal of care	3/3	2/6	3/4	2/2	7/7	7/7	3/3	1/2	0/1	4/5	4/5	4/5	5/6	45/56	N.A.	N.A.
- Survivors at 2 years	5/8 (63%)	7/13 (54%)	6/10 (60%)	10/12 (83%)	14/21 (67%)	11/18 (61%)	6/9 (67%)	13/15 (87%)	8/9 (89%)	9/14 (64%)	11/16 (69%)	6/11 (55%)	10/16 (63%)	116/172 (67%)	N.A.	N.A.
Follow-up at 2 years	5/5	5/7	3/6	9/10	13/14	10/11	6/6	12/13	8/8	8/9	10/11	6/6	9/10	104/116	N.A.	N.A.
NDI	3/5 (60%)	2/5 (40%)	1/3 (33%)	3/9 (33%)	4/13 (31%)	1/10 (10%)	2/6 (33%)	3/12 (25%)	4/8 (50%)	2/8 (33%)	5/10 (50%)	2/6 (33%)	3/9 (33%)	35/104 (34%)	-0.31 [a]	.757 [a]
Favourable outcome	2/5 (40%)	3/5 (60%)	2/3 (67%)	6/9 (67%)	9/13 (69%)	9/10 (90%)	4/6 (67%)	9/12 (75%)	4/8 (50%)	6/8 (67%)	5/10 (50%)	4/6 (67%)	6/9 (67%)	69/104 (66%)	-0.65 [a]	.516 [a]
**PVHI**	25 (8%)	15 (5%)	31 (9%)	17 (49%)	28 (8%)	25 (7%)	15 (4%)	26 (7%)	24 (6%)	20 (5%)	21 (5%)	20 (5%)	23 (5%)	290/4956 (6%)	0.07 [a]	.218 [a]
- Gestational age	26.4 (1.5)	26.5 (1.5)	26.8 (1.7)	26.8 (1.4)	26.7 (1.8)	27.1 (1.8)	26.7 (1.7)	26.1 (1.4)	25.9 (1.5)	26.0 (1.4)	26.5 (1.7)	26.0 (1.4)	26.1 (1.4)	-	-0.17 [b]	.004 [b]
- Death	15/25 (60%)	9/15 (60%)	21/31 (68%)	10/17 (59%)	20/28 (71%)	14/25 (56%)	9/15 (60%)	16/26 (62%)	13/24 (54%)	11/20 (55%)	12/21 (57%)	12/20 (60%)	13/23 (57%)	175/290 (60%)	0.87 [a]	.386 [a]
Withdrawal of care	10/15	7/9	11/21	10/10	18/20	10/14	8/9	14/16	10/13	9/11	11/12	10/12	13/13	141/175	N.A.	N.A.
- Survivors at 2 years	10/25 (40%)	6/15 (40%)	10/31 (32%)	7/17 (41%)	8/28 (29%)	11/25 (44%)	6/15 (40%)	10/26 (38%)	11/24 (46%)	9/20 (45%)	9/21 (43%)	8/20 (40%)	10/23 (43%)	115/290 (40%)	N.A.	N.A.
Follow-up at 2 years	7/10	5/6	4/10	5/7	8/8	10/11	4/6	9/10	8/11	8/9	8/9	7/8	9/10	92/115	N.A.	N.A.
NDI	4/7 (57%)	3/5 (60%)	2/4 (50%)	3/5 (60%)	7/8 (88%)	2/10 (20%)	2/4 (50%)	5/9 (56%)	4/8 (50%)	5/8 (63%)	3/8 (38%)	4/7 (57%)	5/9 (55%)	49/92 (53%)	-0.54 [a]	.588 [a]
Favourable outcome	3/7 (43%)	2/5 (40%)	2/4 (0%)	2/5 (40%)	1/8 (13%)	8/10 (80%)	2/4 (50%)	4/9 (44%)	4/8 (50%)	3/8 (38%)	5/8 (23%)	3/7 (43%)	4/9 (45%)	43/92 (47%)	-1.11 [a]	.265 [a]

[a], Cochran-Armitage test for trend; [b], Univariate linear regression.
