# Supplementary material for: Trends in Outcomes of Major Intracerebral Haemorrhage in a National Cohort of Very Preterm Born Infants in Switzerland
Source: Children (Basel). 2023 Aug 19;10(8):1412. doi: 10.3390/children10081412 (PMC10453192; doi:10.3390/children10081412)
Supplement: Supplementary file 1 [file children-10-01412-s001.zip › Thwaites et al. Table S2.pdf]

Table S2: Neonatal baseline characteristics and demographics of infants visited at follow-up and lost to follow-up.

Mean (SD), N (%)	Infants visited at follow-up N = 196	Infants lost to follow-up N = 35	P value
Male sex	115 (59)	17 (49)	.266
Gestational age (weeks)	27.2 (1.5)	27.4 (1.6)	.573
Multiple birth	38 (19)	6 (17)	.755
Birth weight (grams)	999 (252)	1028 (258)	.586
Z - score	0.18 (0.75)	0.14 (0.75)	.497
Small for gestational age	5 (2)	1 (3)	.916
Completed antenatal steroids course	120 (61)	18 (51)	.276
Chorioamnionitis	27 (14)	1 (3)	.084
Caesarean section	137 (70)	25 (71)	.889
Arterial cord pH	7.27 (0.12)	7.28 (0.16)	.082
10’ Apgar score	8 (7 - 9)	8 (7 - 9)	.755

NEC n (%)	9 (5)	1 (3)	.642
Sepsis n (%)	53 (27)	8 (23)	.605
Oxygen after 36 Weeks PMA n (%)	46 (23)	3 (9)	.047
ROP n (%)	6 (3)	0	.300
Patent arterial duct	112 (57)	18 (51)	.118
Length of hospital stay (days)	77.6 (30.3)	72.1 (29.8)	.891
Family socioeconomic status	6 (4 - 8)	6 (4 – 7.5)	.377

cUS, cranial ultrasound; G3-IVH, grade 3 intraventricular haemorrhage; PVHI, periventricular haemorrhagic infarction; NEC, necrotising enterocolitis; ROP, retinopathy of prematurity; PMA, postmenstrual age; independent Student's *t*- and χ^2 tests as appropriate.
